# “I train and mentor, they take them”: A qualitative study of nurses' perspectives of neonatal nursing expertise and its development in Kenyan hospitals

**DOI:** 10.1002/nop2.442

**Published:** 2020-01-19

**Authors:** Mary Nyikuri, Pratap Kumar, Mike English, Caroline Jones

**Affiliations:** ^1^ Strathmore University Business School Nairobi Kenya; ^2^ KEMRI‐Wellcome Trust Research Programme Nairobi Kenya; ^3^ Nuffield Department of Clinical Medicine Centre for Tropical Medicine and Global Health University of Oxford Oxford UK

**Keywords:** ethnography, experience, LMICs, newborn health, nurses, nursing, qualitative research

## Abstract

**Aims and Objectives:**

Neonatal inpatient care is reliant on experienced nursing care, yet little is known about how Kenyan hospitals foster the development of newborn nursing experience in newborn units.

**Design:**

A Qualitative ethnographic design.

**Methods:**

Face to face 29 in depth interviews were conducted with nurses providing neonatal care in one private, one faith based and one public hospital in Nairobi, Kenya between January 2017 and March 2018. All data were transcribed verbatim, coded in the original language and analysed using a framework approach.

**Results:**

Across the sectors, nurses perceived experience as important to the provision of quality care. They noted that hospitals could foster experience through recruitment, orientation, continuous learning and retention. However, while the private hospital facilitated experience building the public and faith‐based hospitals experienced challenges due to human resource management practices and nursing shortages.

**Conclusion:**

Health sector context influenced how experience was developed among nurses.

**Implications:**

Nurturing experience will require that different health sectors adopt better recruitment for people interested in NBU work, better orientation and fewer rotations even without specialist nurse training.

## 1 INTRODUCTION

The need to reduce neonatal mortality has become a priority internationally, with Sustainable Development Goal (SDG) 3 targeting a reduction in mortality to 12/1,000 live births or lower (WHO, 2016). Kenya has a neonatal mortality rate of 22 per 1,000 live births (Government of Kenya, 2014; WHO, 2016) with 43,600 annual neonatal deaths, the 6th highest in sub‐Saharan Africa (Afolabi, 2017). In many low‐ and middle‐income countries (LMICs), the survival of sick newborns admitted as inpatients is hampered by several factors including poor quality care (Dickson, 2014). Globally and even here in Kenya most inpatient care for sick newborns is provided by nurses (Gathara et al., 2018; Hughes, 2006). Providing care for sick newborns is a complex nursing task that requires experienced nurses (Hill, 2010). Since 2012 in Kenya, a specialist 1 year postbasic training in neonatal nursing was approved by the Nursing Council of Kenya, the body that regulates training and practice of nurses in Kenya.(Murphy et al., 2018) However, the number of nurses who have undergone this training nationally is only approximately 100 nurses. Thus, most nurses providing neonatal care in Kenyan hospitals learn their skills on the job (Murphy et al., 2018). However, little is known about the experiential learning of those providing care or how organizations facilitate or constrain the acquisition of experience to give quality care among their workforce.

## 2 BACKGROUND

Benner's work on the acquisition of experience showed that besides education, hands on experience is at the heart of good clinical judgement (Benner, 1984). Studies in high‐income settings (HIS) have shown that hospital context has a significant influence on nurses’ ability to gain experience (Coles et al., 2017; Kringos et al., 2015; McHugh & Lake, 2010; Tomoaia‐Cotisel et al., 2013).

Experience is developed when nurses are exposed to different patient conditions and clinical scenarios that contribute to the development of knowledge, technical skills and critical thinking, which may influence the effectiveness of nurse surveillance (Audet, Bourgault, Bourgault, & Rochefort, 2018). Karen Hill argues that although a new nursing graduate may have a strong theoretical understanding of nursing, experiential knowledge is essential for them to progress to safer levels of practice (Hill, 2010). She argues that the delivery of quality care depends on nurses who are both well‐educated and experienced (Hill, 2010).

Benner defines experience as both time in practice and self‐reflection that allows preconceived notions and expectations to be confirmed, refined or disconfirmed in real circumstances (Benner, 1984). She identifies a five‐stage process of moving from novice to expert as shown in Table 1. Daley agrees that to be considered an expert in a specific area of nursing nurses should be exposed to events that occur in that clinical setting and judged on how they respond to those events (Mann & De Gagne, 2017).

**Table 1:** From novice to expert; adapted from Benner (1984)

Level of development	Characteristics
Novice	Has no experience of a situation, uses textbook rules which have been learned objectively with no contextual meaning to apply
Advanced beginner	Is able to give marginally acceptable performance can perform a task as asked, but cannot think ahead, change course or prioritize
Competent	Characterized by conscious deliberate planning, able to make long‐range plans, to be efficient and organized
Proficient	Can see beyond the moment, taking in the patients’ total needs and care
Expert	Has an intuitive grasp no longer needs to use analysis or rules. Is able to recognize patterns and quickly make decisions

In this article, we draw on Brenner's definitions and Hill's description that experience is tacit knowledge, “the how to’ knowledge, which is acquired through observations, preceptorship learning opportunities and working with mentors (Hill, 2010). While the existence of the gaps in human resources for health in sub‐Saharan Africa and Kenya are well described (Gupta et al., 2015; MoH, 2012), little is known about the experiential learning of those providing care or how organizations facilitate or constrain the acquisition of experience to give quality care among their workforce. The data for this paper are derived from a broader ethnographic study exploring nurses’ perceptions of what constitutes “quality care” in the neonatal unit. This paper focusses on experience, its importance in facilitating nurses’ ability to give quality inpatient care to sick newborn babies and how different hospital contexts (public, faith based and private) facilitate or constrain the nurses’ acquisition of experience. The research question was therefore “how does one acquire experience in and how does your work environment facilitate the process?”

## 3 METHODS

### 3.1 Design and study setting

This study was a qualitative ethnographic study in three hospitals from public, private and faith‐based offering 24‐hr inpatient care for sick newborns in Nairobi, Kenya. This is because Kenya operates a pluralistic health sector with care services being provided by the Government (public), private for profit and private not for profit (herein referred to as faith based) institutions (Chuma & Okungu, 2011). The public sector gives approximately 50%–60% of the health services, while the remaining health services are provided by the private and faith‐based sectors (Ministry of Health, 2013; Population & MEASURE/DHS+, 2005). This study was undertaken in the Newborn Units (NBUs, the inpatient neonatal care wards) of one public, one faith based and one private hospital, in Nairobi County, Kenya. Table 2 summarizes the key characteristics of NBUs in the study hospitals.

**Table 2:** Characteristics of the neonatal units

Area	Description	Facilities		
		Public	Private	Faith based
Total NBU floor size in square feet	Does not include kangaroo area	1,380	NICU‐ 1,880 NHDU 220	360
Capacity	No. of cots	21	10	0 (encourage contact with mother)
	No. of incubators	11	25	7
Cost of care	Cost for admission only per night in USD	Free but some costs on medications not available in the hospital	160–370	4–10
	Payment method	Out of pocket	Private and national insurance fund	Out of pocket
Staffing	How nurses are allocated	Per shift	Per number of admissions	Per shift
Kangaroo Mother and Father care	Designated area/room	Yes, for mothers and fathers	No	Yes

The study adopted a qualitative ethnographic design (Creswell & Poth, 2017). After obtaining permission from top hospital administrators and NBU ward managers, the primary author created rapport with nurses working in the NBUs by sharing with them a detailed information sheet regarding the study prior to consenting them. It was between January 2017 and March 2018, when twenty‐nine in‐depth interviews using an interview guide were also conducted with nurses who were purposively sampled providing neonatal care, 10 in the public, 8 in the faith based and 11 in the private hospital. All except 3 interviews were digitally recorded using an audio digital recorder. The three interviewees did not consent to be recorded. All available nurses during the study period in the hospital were eligible for participation and except in the private hospital, all available nurses in the public and faith based were interviewed after voluntary consenting. Twenty‐eight interviews were conducted in English while one was conducted in Kiswahili, Kenya's national language. the first author was a PhD student undertaking her research and had no prior knowledge of the nurses.

The findings have been reported using the SRQR checklist. “See Supplementary File 1”.

The research received ethics clearance certificate number SU‐IRB 0060/16/3555 and participating hospitals as well as County Department of Health. All participants were provided written informed consent before participation in interviews.

### 3.2 Data analysis

Analysis began by open coding to understand quality from nurses’ perspectives. Initial codes were generated from the research objectives, but additional codes were developed iteratively as they arose from observation and an initial careful reading of the transcripts. Using Nvivo 10 software for management, we coded the data in their original language and translated into English where necessary (Castleberry, 2014). Emerging themes were discussed across co‐authors while credibility was ensured through feedback to the nurses for sense checking; thirdly, the researcher made presentations in various local forums where feedback was received and incorporated working. The themes identified and presented in this paper are recruitment, orientation, continuous leaning and retention.

## 4 RESULTS

### 4.1 Demographic characteristics of the participants

Twenty‐nine nurses were interviewed, most of whom were aged between 30–39 years and more than half had received training at diploma level. In Kenya, nurse training institutions offer programs at three levels: certificate, diploma, degree (BScN) and Masters (Msc) in nursing. A masters takes two years, a BScN involves 4 years of training followed by a 1‐year internship; a diploma requires 3 years of training while a certificate in nursing can be gained after two and a half years of training but do not involve internship (MoH, 2012). A higher diploma can be gained by nurses who have a basic diploma who have worked for at least two years and involves one year of further study. Diploma level and degree level training involve 2 and 4 weeks of specific training in neonatal care, respectively. Six of the nurses in the private hospital were trained to degree level and one had a Masters. This contrast to the faith‐based hospital where only one nurse had been trained to degree level and the public where there were no nurses in the NBU with degree level training. Table 3 summarizes demographic characteristics of the study sample.

**Table 3:** Demographic characteristics of the study sample

Characteristic	Sector		
	Public	Private	Faith based
Age distribution			
20–29	0	1	4
30–39	4	8	2
40–49	5	2	2
50 and above	1	0	0
Training level			
Diploma	8	4	6
Higher Diploma	2	0	1
Bachelors	0	6	1
Masters	0	1	0
Time in NBU			
Range in months and years	2 months to 8 years	6 months to 7 years	1–18 years
Mode	3.5 years	2.5 years	4 years

### 4.2 Nurses views on experience

When the nurses participating in the study were asked for their thoughts on what constitutes quality care in the NBU, nearly all mentioned a nurse's experience as a key component of the provision of quality neonatal care. Some of the nurses spoke about experience in terms of “having worked with babies before” and “being comfortable in role” attributes which could be seen as “competent” nursing (Benner, 1982). Others spoke more specifically about the ability to recognize patterns of behaviour and emergencies without the need to refer to rules, attributes which align with Benner's category of an “expert” nurse (Benner, 1982):

…When you gain experience caring for small babies, it makes you alert; and by looking at a baby who looks stable, you can tell there is something wrong and you admit the baby… Faith based 02

In the public and faith‐based hospitals the ability to give appropriate care in the face of the daily reality of resource scarcity and high workload was seen as a key attribute of an experienced nurse:

…We improvise; we have the present nurses, especially those are experienced, they will attend to priorities only like babies with breathing problems first… Public 03

… these [experienced] nurses are comfortable in their roles can handle challenges and emergencies despite the number of admissions… Public 05

In the private hospital the nurses mentioned that an experienced nurse was particularly valuable as they were able to lead the nursing care and contribute to decision‐making about the care needs of the neonate; including challenging the treatment practices prescribed by doctors or consultants if they felt they were inappropriate:

An experienced nurse is able to cultivate team work with other health workers, criticize and articulate care (prescribed by doctors or consultants) and just by looking at a baby, they are able to tell the problem… Private 11

### 4.3 How hospitals help/fail to build experience that is needed for quality

#### 4.3.1 Recruitment

According to the participating nurses, building experience begins at recruitment. Ensuring that nurses with the right skills and interest to work with newborns are hired was seen as important for attracting the right applicants; people who are keen to gain or share experience in this particular area of work:

…At recruitment, you advertise for the NBU specifically, deciding which skills are missing on your ward and which specific nurse(s) can complement the team… Private 09

…Even when the advertisements to hire nurses are made, they should specify which departments they are hiring for so that nurses interested in the NBU can be hired … Public 05

Despite the nurses’ views that recruitment was an important step in facilitating the delivery of quality nursing care, it was only in the private hospital that the participants said that nurses were recruited specifically to the NBU and prior experience with newborns was specified as a requirement:

…For staffing standards, one of our minimum standards is that s/he must have worked with newborns before… Private 03

Although the nurses ideally spoke of the need to recruit nurses with prior NBU experience and interest, practically those from the public and faith‐based hospitals said that nurses were recruited generally without specifying their previous experience or interest in working with newborns. Irrespective of how the nurses ended up in the NBU, the participants across the sectors described three processes that they perceived as important in creating an experienced nurse. These were orientation; continuous learning; and retention.

#### 4.3.2 Orientation

The orientation process was used to introduce the nurses to their environment, an induction onto the ward. However, from observations what was covered in orientation differed across settings. In the public, it was to familiarize with the setting by getting to know the layout, the team composition, location of different equipment and consumables, understand shift times and learn how to calculate fluids and drugs for different conditions. In the faith based, the new nurse was considered an extra hand, not allocated duties and expected to observe what the “old” nurse was doing, ask questions and learn from the colleagues all they needed to know to understand their workspace and clinical challenges in the newborns. In the private sector, there was a formal orientation tool which guided what a new nurse had to cover each day. There were different nurses allocated to orient the new nurse on the layout, team composition, shift processes and times, where to get tea and lunch as well the routines of the ward. All nurses mentioned orientation as an essential process that helped build experience for quality care delivery:

When a nurse comes to the NBU, they need orientation, what others know should be passed onto them… Public 01

When there is a new staff, before they venture into treating the critical patients, they go through orientation to enable her be at par with the rest … Private 09

No nurse should be allowed into NBU until they have undergone orientation…during this time they should not be allocated any duties… Faith based 03

Perceptions about the length of time required for orientation varied across the sectors, perhaps mirroring the practical realities in each setting. Nurses in the public sector reported that orientation for a new nurse to gain an understanding of the ward and her role, should last 2–3 days:

A new nurse to the ward is given orientation for about 2‐3 days which are good to help them understand their roles… Public 09

The faith‐based hospital nurses said that orientation should last for a week:

For a week, they have to watch and learn, before we cannot allocate them duties… Faith based 03

By contrast, in the private hospitals the nurses said that two weeks’ orientation was required for permanent staff and a week for locums. However, these locums were re‐oriented if they had taken more than three months before being engaged on the ward because of what they attributed to differences in context:

So once new nurses and even locums are picked they come for two weeks and a week respectively for orientation… because they may come once a week at times not at all in 3 months so it is like all the time they come they are like new need orientation… Private 10

While all the participating nurses emphasized the importance of a period of orientation, some of the nurses (4/10) in the public sector hospital said that they had started working on the NBU with no orientation. They perceived that their lack of orientation had been due to staff shortages and the lack of processes to ensure systematic implementation. One of the nurses who had not received orientation and was starting as a novice in the NBU environment said that she had experienced frustration as she did not know where to get equipment and medications and she had to refer to charts on the wall to conduct routine tasks such as how to calculate feeds and medications:

Ok, orientation depends on who you find on the ward. If you find a few nurses when you are reporting here, you will have to know where things are kept by yourself and keep reading the protocols on the walls to know what to do…I had the worst experience, I came when it was a change over for everybody so we were all new, except the in‐ charge, I was allocated morning off alone first day in the ward…. I was so stressed but used my phone to consult with the in‐charge on how to mix fluids… Public 10

All nurses who underwent the orientation were appreciative:

Yes, the ward in charge showed me acute rooms and what happens in each cubes, stationeries, drugs, store… Public 05

…they take you through orientation to understand the environment where and what you need is… Private 02

After one week of orientation, I was comfortable enough to work alone. It makes it easy for the new staff to be integrated into the system… Faith based 08

From informal discussions and observations, the intention of orientation is to introduce the novice nurses in the public and faith based to their work environment and allow then to learn how different disease conditions manifest in babies. For the private hospital, the nurse is recruited with some prior experience, therefore, their orientation focusses on moving them to an advanced beginner. Once on the ward the nurses have opportunities to receive mentorship from expert nurses. I observed that across facilities, the in charge tried to allocate less experienced nurses on shifts where they could work under the guidance and mentorship of more experienced nurses.

#### 4.3.3 Continuous learning (CL)

Following on from orientation, around half of the nurses from each hospital mentioned that opportunities for continuous learning were important for acquiring neonatal nursing‐specific skills. The opportunities described were in the form of short courses, seminars, continuous medical education (CMEs) e‐learning and workshops:

…Nurses need some more small trainings as things change, for example when I attended an Emergency Triage Assessment and Treatment (ETAT), I realised some things have changed … Public 05

Continuous learning with constant updates should not be taken for granted, because everything is changing and in order to stay above, they should invest in staff… Private 10

There needs to continuous updates ‐ in form of seminars to educate nurses on new standards… Faith based 05

In the private hospital attendance at CL sessions was mandatory as attendance formed part of their nursing performance reviews. Attendance at CL sessions was not mandatory in either the public or the faith hospitals but attendance at such sessions was taken into consideration when a nurse applied for long‐term training opportunities and for promotion. It was observed nurses in both public and private attend CMEs on Kangaroo Mother Care and resuscitation, respectively. However, during the three months of observation in the faith‐based hospital no opportunities to attend CL sessions were observed.

While ideally, all three hospitals had different opportunities for continuous learning, in practice few nurses in the public or faith‐based hospitals were able to take up these opportunities. This was attributed to staffing shortages on the ward making it difficult to attend:

CMEs are there but due to shortage we don’t get time to attend… Public 08

In the faith‐based hospital, one nurse mentioned that they (junior staff) were not given the opportunity to attend training, instead a member of senior management would attend the sessions and subsequently give occasional updates to those who had not been able to attend:

…From 9th there is a seminar in large hospital where we may have two people from management representing … Faith based 04

In addition to face‐to‐face CL sessions, opportunities for learning new skills were available to the nurses in the private hospital through their access to computers and the Internet allowing them to update themselves on the latest guidelines and care procedures during personal time. The nurses in the public and faith‐based hospitals did not have access to computers and the Internet which hampered personal e‐learning for the nurses in these hospitals:

We have no computers and internet to update themselves on latest care ‐ but I blame it on old school mentality… Faith based 05

#### 4.3.4 Retention on the ward

Another avenue through which experience in neonatal nursing is built is by retaining experienced nurses in the NBU. Retention was mentioned as being a motivator for nurses to sharpen their skills in neonatal care. The advantages of staying on the NBU were articulated by one of the participants from the private hospital who said:

I like the specialty, no mixing pediatrics and newborns, when you are neonate you are neonate… Private 03

A nurse in the public hospital mentioned that nurses in the NBU were not keen on investing in a diploma in neonatal nursing partly because they feared being taken to other wards where their skills could be redundant:

…It is quite some investment… I paid for myself…but others fear because they can be moved to other wards… Public 01

Another participant from the faith‐based hospital emphasized the importance of staying in an NBU to building experience and specific neonatal nursing skills:

When you work here for long, you can see a baby who looks stable, but deep inside, there is something that tells you to admit, this is not taught in college, it is from experience… Faith based 02

Retention of experienced nurses varied. In the public hospital, it was observed that there was a tendency to carrying out frequent change overs (rotation) of staff except for the ward in charge and the deputy. These changeovers were described by most nurses as a hospital strategy to enable nurses to gain experience across different service areas. However, its frequency was described as too often, a strategy that was being used to deal with staffing shortages across wards. It is these frequent change overs that were described by all ten nurses as counterproductive and demotivating:

I train and mentor, they take them…I am frustrated and tired…who has time to waste on nurses who will be taken elsewhere after a few months… Public 03

For the private and faith based, retention challenges which were not common were described as being a result of the desire for nurses to pursue other growth opportunities. Two nurses in the private described the opportunities available for pursuing a degree in nursing as they continued with work as a strategy that helps them stay. However, given opportunities in the national government run hospital they could leave:

…Okay, the opportunity to upgrade to a degree while still on payroll is a plus because then we tend to stay on…however, if I get greener pastures in a parastatal, I would leave…. Private 08

Nurses who expressed a desire to leave attributed it to a desire to earn a higher salary:

…Salaries here are soo small, but again you will be told that even what we charge our patients is little…so, we work as we look for better job opportunities… Faith based 05

## 5 DISCUSSION

Literature from high‐income settings suggests that nurses acquire experience in practice through a variety of ways. Reflection is recognized as an effective means by which nurses can develop their practice (Asselin, 2011; Asselin & Fain, 2013; Tsingos‐Lucas, Bosnic‐Anticevich, Bosnic‐Anticevich, Schneider, & Smith, 2016)other ways include shadowing of experienced nurses and other health professionals on the ward, coaching, supervision and mentorship from existing experienced nurses; seeking clarity regarding the role; being given the opportunity to develop key knowledge and skills to meet their role (Jackson et al., 2015; Muleya, Marshall, Marshall, & Ashwin, 2015; Takase, 2013). In addition, developing expertise has been found to depend on the educational and experiential composition of other staff working in the ward, education level and years of experience of the nurse as well as the context where the nurse practices which may help or hinder expertise development by establishing a culture of professional nursing that encourages, values and gives opportunities for the development of nursing expertise (McHugh & Lake, 2010).

In the Kenyan context, although all nurses employed by the public and private sector are required to renew their license every three years, each health sector has its own nursing practice and workforce management system(Wakaba et al., 2014; Waters et al., 2013). Whereas in the public sector nurses are recruited and managed centrally by the County department of health, the faith based and private sectors have hospital‐based recruitment and management system. There is also a diversity on how different counties plan for and manage their nursing workforce (Wakaba et al., 2014). Findings from this study have shown that nurses who start working in the NBUs in the faith based and public hospitals are likely to be “novices” in that environment (although potentially “expert” in other areas of clinical care) but in the private they are already at least competent. In public and faith based, there are few opportunities for a nurse to develop “expertise” except through practice as there is little CL. While the nurses in the faith based might have the opportunity to become “experts” through continuous practice (although not CME), this is not the case for the public as these nurses are moved across different departments in the hospital.

There is some evidence from low‐ and middle‐income countries (LMICs) in the accountability literature to show that nurses posted as facility managers gain managerial experience through on job training, supportive supervision from their managers and peer learning (Nyikuri, Tsofa, Tsofa, Barasa, Okoth, & Molyneux, 2015). There is also anecdotal evidence to suggest that nurses gain experience in LMICs through attachment, internship and volunteer schemes in private owned clinics and hospitals as well as in government‐owned hospitals. However, no studies were found on how nurses move from being novice to expert in their clinical role and to our understanding, this study is unique in exploring the role of hospitals in LMICs in developing nursing experience for quality care. Previous research in the USA, Australia, UK and Finland has shown that nurses develop substantial knowledge of the strengths and weaknesses of hospital systems through experience (Debono & Braithwaite, 2017; Johnson, 2015; Nielsen, Lasater, Lasater, & Stock, 2016). A systematic review of studies that have applied the Benner theory between 2006–2016 showed that the theory has been applied widely in high‐income countries in exploring nursing practice, nursing education and nursing research (Oshvandi et al., 2016). Most recently, it has been applied and found relevant in understanding the role of nursing experience in clinical decision‐making in acute care settings (Stinson, 2017).

Findings from this study on the importance of development of experience confirm recent findings of a study carried out by a team at London School of Hygiene and Tropical Medicine (LSHTM) who concluded that for LMICs where specially trained neonatal nurses may not be available in all facilities, minimizing regular staff rotation from the neonatal unit may be one of the important aspects of skill and experience building (Moxon et al., 2018). At the ward and hospital levels, nursing leaders need to anticipate dynamism in nurses personal interests and goals and give a positive environment that supports them to decrease nursing turnover rates (Spivak, Smith, Smith, & Cynthia Logsdon, 2011). A study to understand registered nurses (RNs)’ decisions to remain or leave their workplaces in two private hospitals in Kenya showed that among other factors, feelings of non‐appreciation due to lack of the professional development opportunities and poor remuneration influenced their turnover (Mbuthia, Brownie, Brownie, & Holroyd, 2017).

Benner's theory was found to be useful in curriculum development in education institutions (Altmann, 2007). Considering the stages in acquiring expertise as outlined in Benner's theory, more purposeful strategy is argued for developing expertise and therefore quality of NBU nursing care. The lessons gained from this process can be applied to other specialized parts of hospitals. At present most HRH focus is on production of numbers but not on expertise, the global debate needs to include expertise.

### 5.1 Limitations

These study findings are limited; firstly, the study was conducted in a small number of places in one city, but it offers detailed work and triangulation of interviews and observations. Secondly, the authors did not start out by looking at expertise but emerged as an important finding and so interviews and observations did not consider mentorship and supervision on the job but offers insights into area for new research.

## 6 CONCLUSION

Nurses as the backbone of the healthy system globally and in Kenya consider experience in caring for sick newborns as a key aspect in delivering quality care. Benner's theory is useful in understanding how to begin to cultivate and nurture experience. Nurturing experience will require that different health sectors adopt better recruitment for people who wish to work in NBU, better orientation and fewer rotations as key things even without specialist nurse training.

## 7 RELEVANCE TO CLINICAL PRACTICE

These findings are relevant in the clinical practice as they bring to the fore the need for hospitals to support nurses in their efforts to deliver quality care.

First, the findings in this study point to the factor that no matter the sector of practice, nurses perceive that experience is important to their ability to give quality care. Experienced nurses are more likely to be confident to take care of patients as they are comfortable in making clear patient care decisions.

Second, experience in important for nurses to develop positive attitude in the workplace and to guide and nurture it among novices. Third, nurturing experience is an essential and paramount for hospital work environments that are keen on supporting nurses give quality care. This study has identified that health managers need to adopt better recruitment for people who wish to work in NBU, better orientation and fewer rotations as key things even without specialist nurse training.

## CONFLICT OF INTEREST

The author(s) declare that they have no conflict of interests.

## AUTHORS' CONTRIBUTIONS

MN was responsible for conceptualization of the idea, data collection, analysis and write up of initial draft. PK, CJ and ME gave overall support in the analysis and critique of the final paper.

## ETHICS APPROVAL AND CONSENT TO PARTICIPATE

The research received ethics clearance from the Strathmore University and KEMRI certificate no. KEMRI/SERU/CGMR‐C/SU‐IRB 0060/16/3555 and was also approved by the three participating hospitals as well as Nairobi City County department of Health.

## Data Availability

The data that support the findings of this study are available from KEMRI‐Wellcome Trust but restrictions apply to the availability of these data, which were used under license for the current study, and so are not publicly available. Data are, however, available from the authors upon reasonable request and with permission of KEMRI‐Wellcome Trust.
